# Antifungal Activity of 14-Helical β-Peptides against Planktonic Cells and Biofilms of *Candida* Species

**DOI:** 10.3390/ph8030483

**Published:** 2015-08-13

**Authors:** Namrata Raman, Myung-Ryul Lee, David M. Lynn, Sean P. Palecek

**Affiliations:** 1 Department of Chemical and Biological Engineering, University of Wisconsin-Madison, 1415 Engineering Drive, Madison, WI 53706, USA; E-Mail: nraman2@wisc.edu; 2 Department of Chemistry, University of Wisconsin-Madison, 1415 Engineering Drive, Madison, WI 53706, USA; E-Mail: mrlee2@wisc.edu

**Keywords:** *Candida albicans*, *Candida glabrata*, *Candida tropicalis*, *Candida parapsilosis*, biofilms, β-peptide, antimicrobial peptide, antifungal, hydrophobicity

## Abstract

*Candida albicans* is the most prevalent cause of fungal infections and treatment is further complicated by the formation of drug resistant biofilms, often on the surfaces of implanted medical devices. In recent years, the incidence of fungal infections by other pathogenic *Candida* species such as *C. glabrata*, *C. parapsilosis* and *C. tropicalis* has increased. Amphiphilic, helical β-peptide structural mimetics of natural antimicrobial α-peptides have been shown to exhibit specific planktonic antifungal and anti-biofilm formation activity against *C. albicans in vitro*. Here, we demonstrate that β-peptides are also active against clinically isolated and drug resistant strains of *C. albicans* and against other opportunistic *Candida spp*. Different *Candida* species were susceptible to β-peptides to varying degrees, with *C. tropicalis* being the most and *C. glabrata* being the least susceptible. β-peptide hydrophobicity directly correlated with antifungal activity against all the *Candida* clinical strains and species tested. While β-peptides were largely ineffective at disrupting existing *Candida* biofilms, hydrophobic β-peptides were able to prevent the formation of *C. albicans*, *C. glabrata*, *C. parapsilosis* and *C. tropicalis* biofilms. The broad-spectrum antifungal activity of β-peptides against planktonic cells and in preventing biofilm formation suggests the promise of this class of molecules as therapeutics.

## 1. Introduction 

*Candida albicans* is the most common cause of fungal infections in humans, with a high mortality rate of 30%–60% associated with systemic *Candida* infections [1,2]. *C. albicans* infections can be life-threatening in immune compromised individuals, such as those suffering from AIDS or cancer, and organ transplant recipients on immunosuppressive drugs [3,4,5,6,7,8,9]. Further, candidemia, the presence of *Candida* in the bloodstream, is often associated with the presence of an indwelling medical device such as a central venous catheter, cardiac pacemaker, urinary catheter, or orthopedic implant [1,4,10,11]. *C. albicans* form biofilms on the surfaces of these devices and serve as a reservoir of infectious cells [12,13,14]. While there are drugs that are active against *C. albicans*, they act on few specific molecular targets, and drug-resistant *C. albicans* strains lead to reduced efficacy of these drugs in certain cases [15,16]. Further, many of these drugs exhibit decreased effectiveness against *C. albicans* biofilms [17,18]. 

Motivated by the need to develop novel antifungal therapies targeting essential organelles or pathways within the pathogen that cannot easily be bypassed through cell mutations, antimicrobial peptides (AMPs), also known as host defense peptides, have been investigated as potential antifungal drugs [19,20]. While these compounds target a vital component of the cell, the cell membrane, these native peptides exhibit low stability and activity in physiological media and are susceptible to proteolytic degradation *in vivo* [21,22]. Inspired by these naturally occurring AMPs, various groups have designed synthetic analogues that retain or improve the antimicrobial membrane disrupting activities of natural AMPs but possess improved physiological stability *in vitro* and *in vivo* [23,24,25,26]. One class of such molecules is helical oligomers of β-amino acids. These β-peptides have shown promise as antibacterials and antifungals [27,28,29,30].

We have demonstrated that certain 14-helical β-peptide structural features, specifically global amphiphilicity with an intermediate hydrophobicity, are essential for specific antifungal activity against planktonic *C. albicans* [29,31]. However, it is unclear what relationship exists, if any, between the structure of 14-helical β-peptides and their activity against *C. albicans* biofilms, which are more prevalent and difficult to treat in the context of medical device-associated infections. Further, the activity of 14-helical β-peptides has not been investigated in other *Candida spp*. While *C. albicans* is the predominant causative agent of fungal infections in humans, the number of other *Candida* species causing fungal infections is on the rise [4,7,32,33]. Specifically, *C. glabrata*, *C. parapsilosis* and *C. tropicalis* are increasingly becoming among the most commonly isolated pathogens causing fungal infections [33,34,35,36,37]. These three species are also capable of forming biofilms to varying extents and the presence of biofilms is a virulence factor that has been associated with increased mortality during infection [32,38]. 

In this study, we used structural features of α-helical AMPs as a guide to design 16 globally amphiphillic 14-helical β-peptides with approximately three residues per turn. β-peptides can adopt a number of different secondary structures, including the 14-helix, which consists of 14-membered rings stabilized by hydrogen bonding between the O of the C=O at the *i* position and the H in the backbone H-N at the *i*-*2* position [28,39]. These peptides were comprised of 9 or 10 β-amino acids, exhibited a net charge of +4, and contained at least one helix-stabilizing cyclic aminocyclohexane carboxylic acid (ACHC) residue. We demonstrate that a direct correlation exists between β-peptide hydrophobicity, as measured by RP-HPLC retention times, and planktonic antifungal activity against multiple clinical strains of *C. albicans* and against *C. glabrata*, *C. parapsilosis* and *C. tropicalis*. The mechanism of action of these β-peptides is thought to involve membrane disruption [30], and here we demonstrate that the activity of the β-peptide was independent of the presence of the cell wall in *C. albicans*. Our results indicate that β-peptide hydrophobicity has little effect on disruption of existing *Candida spp*. biofilms. However, β-peptides prevented the formation of biofilms in a hydrophobicity-dependent manner. Taken together, our results demonstrate that globally amphiphilic 14-helical β-peptides exhibit activity against the most prevalent fungal pathogens and prevent the formation of biofilms by these organisms, suggesting that these β-peptides have promise as antifungal agents. 

## 2. Results 

### 2.1. Design and Synthesis of 14-Helical β-Peptides 

**Figure 1:**
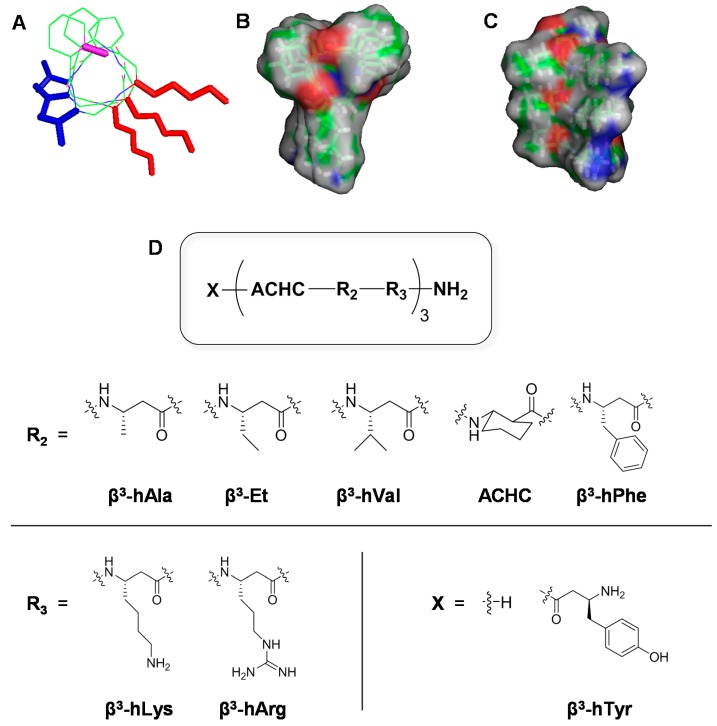
14-Helical β-peptide design and chemical structures. 3D structures (**A**, **B** and **C**) were generated based on available crystal structure data and then geometry was optimized using Gaussian 03 at the B3LYP/6-31G level. (**A**) Stick view of β-peptide **4**. The N-terminus (pink), hydrophobic side chain (blue), and cationic side chain (red) are indicated in color. (**B**, **C**) Surface views of β-peptide **4**. Surface colors represent atom type H (gray), O (red), and N (blue). (**D**) Chemical structures of β-peptides containing a helix-stabilizing ACHC residue and the N-terminus (X), hydrophobic (R_2_), and cationic residues (R_3_) were altered as indicated to vary peptide hydrophobicity.

We designed and synthesized a set of sixteen 14-helical β-peptides, **1**–**16**, (Figure 1, Table 1) that are 9 or 10 β-amino acid residues long and possess a net charge of +4. Based on our previous work demonstrating the importance of amphiphilicity and helix stability in β-peptide antifungal activity [29,30,31], all β-peptides used in this study were designed to be globally amphiphilic and contained approximately three residues per helical turn with at least one helix-stabilizing ACHC residue in every turn. To elucidate β-peptide structure-function relationships, we changed the structures of the peptide by varying: (i) the absence (**1**–**8**) or presence (**9**–**16**) of an N-termimal β^3^-hTyr residue, (Figure 1, Table 1, X), (ii) the hydrophobic residue (Figure 1, Table 1, R_2_) as β^3^-hAla, β^3^-Et, β^3^-hVal, ACHC, or β^3^-hPhe, and (iii) the cationic residue of the helical repeat to be either β^3^-hLys or β^3^-hArg (Figure 1, Table 1, R_3_). These structural variations also affected the β-peptide hydrophobicity as measured by RP-HPLC (Table1). 

β-peptides were produced by microwave-assisted Fmoc synthesis at 20−40 μmol scales and purified by RP-HPLC using a C18 column. MALDI mass spectrometry was used to validate the mass of each peptide as described previously [31]. Retention times determined by C18 RP-HPLC were used as a measure of the relative hydrophobicity of the β-peptides (Table 1). This approach has been used previously by our group and others to assess the relative hydrophobicity of different peptide structures [31,40,41]. Preliminary antifungal activity and mammalian toxicity evaluation were reported previously for β-peptides **1**–**16** as planktonic MIC against *C. albicans* and percent hemolysis at the MIC, respectively [31]. Concentrations of β-peptide resulting in 50% hemolysis (HC_50_) for β-peptides **1**–**16** are also provided in Table S1. 

### 2.2. Planktonic Antifungal Activity of β-Peptide Is a Function of Hydrophobicity in Multiple C. albicans Strains

Antifungal activity of the β-peptides against planktonic *C. albicans* was measured by quantifying the minimum inhibitory concentration (MIC) of the peptides according to a modified version of the protocol prescribed by the Clinical Laboratory Standards Institute (CLSI). We evaluated the planktonic antifungal activities of peptides **1**–**8** against three different *C. albicans* clinical isolates: SC5314, ATCC 90028, and K1. The K1 strain, isolated from a systemic *Candida* infection, forms dense biofilms and is also fluconazole-resistant [42,43,44]. Table 2 shows the RP-HPLC retention times, as a measure of hydrophobicity, and the MICs of the β-peptides against the three different clinical strains. The corresponding quantitative XTT measures of *C. albicans* metabolic activity as a function of β-peptide concentration are provided in Figure S1. MICs of the β-peptides varied from 4 µg/mL to greater than 128 µg/mL, the highest concentration of each peptide tested. We observed very little to no variation in the MIC values across different *C. albicans* strains for all the β-peptides. Further, an inverse correlation existed between the HPLC retention times of the β-peptides and their MICs against all *C. albicans* strains tested, with β-peptide **8** being the most hydrophobic and active, followed by β-peptides **5** and **4**. 

**Table 1:** Sequence and RP-HPLC retention times of 14-helical β-peptides, **1**–**16**, used in this study.

Peptide #	N-Terminus ^a^ (X)	Hydrophobic Residue ^a^ (R2)	Cationic Residue ^a^ (R3)	Hydrophobicity (HPLC Retention Time, min) ^b^
1	H	β3-hAla	β3-hLys	19.3 ± 0.1
2	H	β3-Et	β3-hLys	22.5 ± 0.2
3	H	β3-Et	β3-hArg	23.2 ± 0.1
4	H	β3-hVal	β3-hLys	24.5 ± 0.2
5	H	β3-hVal	β3-hArg	25.4 ± 0.1
6	H	ACHC	β3-hLys	23.1 ± 0.2
7	H	ACHC	β3-hArg	23.8 ± 0.1
8	H	β3-hPhe	β3-hLys	26.2 ± 0.2
9	β3-hTyr	β3-hAla	β3-hLys	20.4 ± 0.2
10	β3-hTyr	β3-Et	β3-hLys	23.5 ± 0.1
11	β3-hTyr	β3-Et	β3-hArg	24.2 ± 0.1
12	β3-hTyr	β3-hVal	β3-hLys	25.7 ± 0.1
13	β3-hTyr	β3-hVal	β3-hArg	26.5 ± 0.2
14	β3-hTyr	ACHC	β3-hLys	24.0 ± 0.2
15	β3-hTyr	ACHC	β3-hArg	24.6 ± 0.2
16	β3-hTyr	β3-hPhe	β3-hLys	27.4 ± 0.2

^a^ All three-letter amino acid codes refer to β^3^-homoamino acids that have the same side chain as the corresponding α-amino acids. β^3^-Et refers to an ethyl side chain in the β^3^ position and ACHC refers to *trans*-2-aminocyclohexanecarboxylic acid; ^b^ Error denotes standard deviation of triplicate experimental measurements.

**Table 2:** Minimum inhibitory concentrations (MICs) of peptides **1**–**8** against *C. albicans* clinical isolates.

Peptide #	RT ^a^ (min)	MIC ^b^ (µg/mL)		
		ATCC90028	K1	SC5314
**1**	19.3	>128	>128	>128
**2**	22.5	64	64	64
**6**	23.1	32	32	16
**3**	23.2	32	32	32
**7**	23.8	32	16	16
**4**	24.5	16	16	8
**5**	25.4	16	8	8
**8**	26.2	8	4	8

^a^ The average value obtained from three independent analytical RP-HPLC measurements; ^b^ MICs were determined by taking the average of three experiments of three replicates each. *C. albicans* cells (10^3^ cells/mL) were incubated with β-peptides for 48 h and β-peptide susceptibility was assessed using an XTT reduction assay to compare the absorbance at 490 nm for β-peptide-treated samples and untreated samples.

### 2.3. β-Peptides Kill Planktonic C. glabrata, C. parapsilosis and C. tropicalis Cells in a Hydrophobicity-Dependent Manner

**Figure 2:**
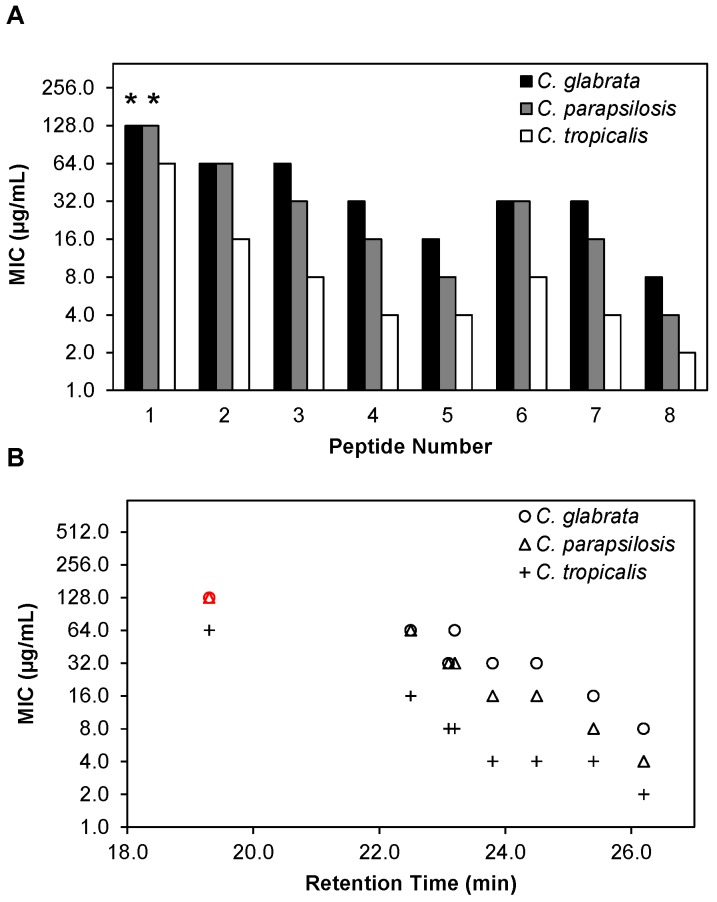
β-peptides are effective against planktonic *C. glabrata*, *C. parapsilosis*, and *C. tropicalis* and antifungal activities correlate with β-peptide RP-HPLC retention times. (**A**) Minimum inhibitory concentrations (MICs) of peptides **1**–**8** against *C. glabrata* (black bars), *C. parapsilosis* (grey bars), and *C. tropicalis* (white bars). MICs were determined by incubating *C. glabrata*, *C. parapsilosis*, and *C. tropicalis* cells (10^3^ cells/mL) with β-peptides for 48 h and β-peptide susceptibility was assessed using an XTT reduction assay to compare the absorbance at 490 nm for β-peptide-treated samples and untreated samples. (**B**) Plot of planktonic MIC versus RP-HPLC retention times of peptides **1**–**8** against *C. glabrata* (circles), *C. parapsilosis* (triangles), and *C. tropicalis* (plus signs). Experiments were performed on at least two independent days with three replicates each. Asterisks (*) and red symbols indicate that MIC was > 128 µg/mL, the highest concentration of peptide assayed.

Relatively few AMPs have been investigated for their activity against *Candida* species other than *C. albicans* [45,46,47]. We hypothesized that because of their membrane disruption-based mechanism of antifungal activity [30,48,49], β-peptides might also be active against other *Candida* pathogens*.* We therefore performed planktonic susceptibility experiments against *C. glabrata*, *C. parapsilosis* and *C. tropicalis* according to a modified protocol of CLSI, with a quantitative XTT end point (Figure 2A and Figure S2), similar to susceptibility experiments performed on *C. albicans* described above. The set of β-peptides **1**–**8** exhibited a wide range of MICs against all three species, from 8 µg/mL to greater than 128 µg/mL against *C. glabrata*, from 4 µg/mL to greater than 128 µg/mL against *C. parapsilosis*, and from 2 µg/mL to 64 µg/mL against *C. tropicalis* (Figure 2A, Table S2). In general, for all β-peptides tested, *C. tropicalis* was most susceptible to the peptide with comparatively lower MICs and *C. glabrata* was the least susceptible to the β-peptide with relatively higher MICs. 

We next investigated the influence of β-peptide hydrophobicity on activity against *C. glabrata*, *C. parapsilosis* and *C. tropicalis*. We observed a similar inverse relationship between the hydrophobicity of the β-peptide and the MIC against all three pathogens (Figure 2B). Overall, β-peptide **8**, the most hydrophobic β-peptide tested, was the most active against all *Candida* species tested, while β-peptide **1**, the least hydrophobic, was the least active against all species. 

**Figure 3:**
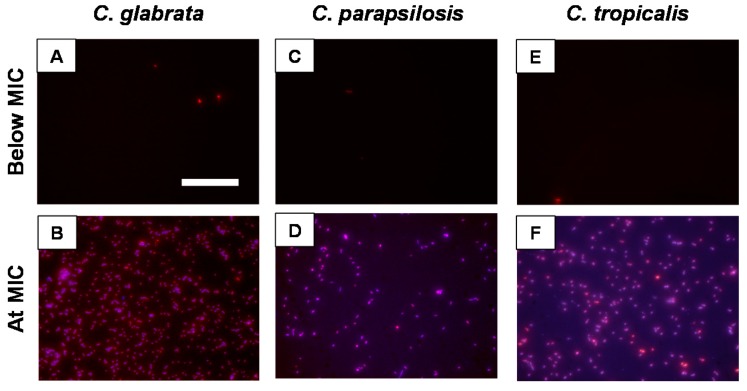
Fluorescence micrographs of *C. glabrata* (**A**, **B**), *C. parapsilosis* (**C**, **D**), and *C. tropicalis* (**E**, **F**) treated with peptide **4_FL_**. Cells (10^5^ cells/mL) were treated with peptide **4_FL_** (blue) at a concentration 4-fold below the MIC (**A**, **C**, **E**) and at the MIC (**B**, **D**, **F**) for 3.5 h. Cells were stained with PI to identify dead cells (red). Scale bar = 100 µm.

To corroborate the MIC data based on XTT measurements, we performed a fluorescence assay to visualize the colocalization of β-peptide with dead cells in different *Candida spp*. We selected peptide **4**, which had a relatively low MIC against all *Candida spp*., and is also specific *i.e.*, non-toxic to mammalian cells with a relatively high HC_50_ of 161 µg/mL (Tables S1and S2) and labeled it with coumarin (peptide **4_FL_**) as described previously for monitoring β-peptide localization on *C. albicans* cells [30]. We verified that the addition of the fluorescent label to the peptide did not substantially affect the MIC of the β-peptide (Table S3). In all *Candida* species at concentrations up to 4-fold below the MIC, very few dead cells were detected by PI staining (Figure 3 and Figure S3). At the MIC and at higher concentrations of the β-peptide, we observed substantial cell death by PI staining, with the labeled β-peptide associated with the cells (Figure 3 and Figure S3). 

### 2.4. The Cell Wall Does Not Significantly Affect the Activity of β-Peptides against C. albicans 

**Figure 4:**
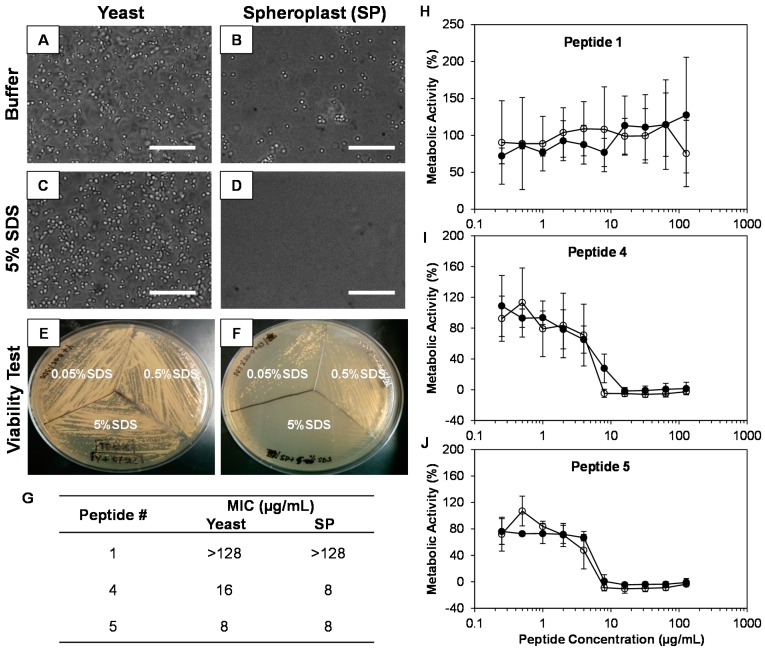
β-peptide activity against *C. albicans* spheroplasts (SPs). (**A**–**D**) Phase contrast microscopy of overnight cultures of yeast (**A**, **C**) or after cell wall removal to yield SPs (**B**, **D**) in buffer solution (**A**, **B**) and in the presence of 5% SDS (**C**, **D**). Scale bars = 50µm. Agar plates showing viability of yeast (**E**) and spheroplasts (**F**) in the presence of increasing concentrations of SDS. (**G**) Comparison of the MICs of *C. albicans* yeast and SPs against β-peptides. (**H**–**J**) Plots of concentration-dependent growth inhibition of *C. albicans* yeast (black circles) and SPs (white circles) by β-peptides **1**, **4** and **5**. *C. albicans* yeast cells and SPs (10^3^ cells/mL) were incubated with β-peptides for 48 h and β-peptide susceptibility was assessed using an XTT assay to compare the absorbance at 490 nm for β-peptide-treated samples and untreated samples. Data points are the average of two independent experiments of three replicates each and error bars denote standard deviation.

The mechanism of action of β-peptides against *C. albicans* is understood to be through the disruption of the cell membrane [30]. Here, we evaluated if the *C. albicans* cell wall affects the activity of the β-peptides. We enzymatically degraded *C. albicans* cell walls to generate spheroplasts (SPs). We confirmed the generation of SPs using visual observation by microscopy (Figure 4A–D) and by evaluating the susceptibility of SPs to increasing concentrations of SDS. SPs were more susceptible than yeast cells to SDS, with complete SP disruption observed at 0.5% SDS while yeast cells remained intact (Figure 4E,F). We then performed planktonic susceptibility assays of freshly prepared SPs and yeasts, in the presence of varying concentrations of β-peptide to evaluate the MIC of the peptide against SP and yeast, to elucidate the influence of the cell wall on MIC. We observed no significant differences in MIC values against either SPs or yeast for active β-peptides **4** and **5** and for inactive β-peptide **1** (Figure 4G–J).

### 2.5. β-Peptide Hydrophobicity Does Not Affect Activity against Existing C. albicans Biofilms 

**Figure 5:**
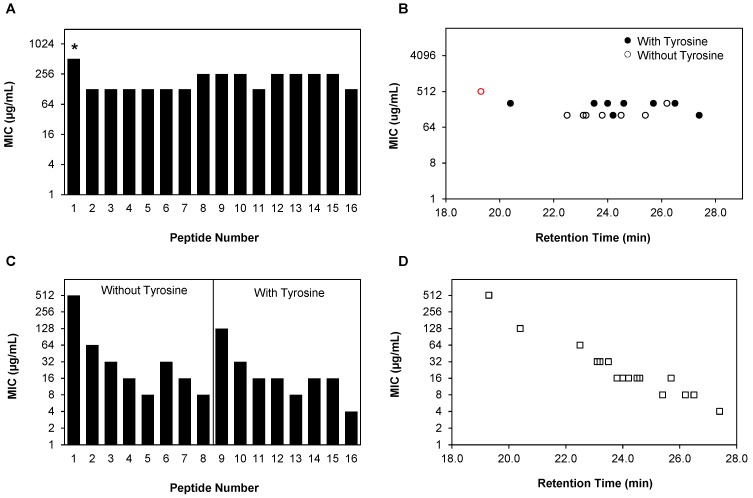
Antifungal activities of β-peptides against *C. albicans* biofilms. (**A**) Mature biofilms were formed by incubating *C. albicans* cells (10^6^ cells/mL) for 48 h. Planktonic cells were removed, biofilms were washed, and 2-fold dilutions of β-peptides were added and incubated for an additional 48 h. β-peptide MICs were quantified using an XTT assay to compare the absorbance at 490 nm for β-peptide-treated samples and untreated samples. (**B**) Plot of biofilm MICs versus RP-HPLC retention times of peptides **1**–**16** against *C. albicans* for peptides without (white circles, peptides **1**–**8**) and with (black circles, peptides **9**–**16**) an N-terminal *β^3^*-hTyr residue. (**C**) MICs against *C. albicans* biofilm formation were determined by incubating *C. albicans* cells (10^6^ cells/mL) with 2-fold dilutions of β-peptides for 48 h. Planktonic cells were removed, biofilms were washed and β-peptide susceptibility was assessed using an XTT assay to compare the absorbance at 490 nm for β-peptide-treated samples and untreated samples. (**D**) Plot of MICs to prevent biofilm formation versus RP-HPLC retention times of the peptides **1**–**16** against *C. albicans.* Experiments were performed on at least two independent days with three replicates each. Asterisks (*) and red symbols indicate that MIC was > 512 µg/mL, the highest concentration of peptide assayed.

We next investigated whether β-peptides were effective against biofilms formed for 48 h in the absence of β-peptide, and to what extent hydrophobicity of the β-peptide affected the MIC against these biofilms. We evaluated two different series of peptides of increasing hydrophobicity, peptides **1**–**8** and **9**–**16**, that were synthesized without and with an N-termimal β^3^-hTyr residue (Table 1, Figure 5A and Figure S4). MICs against existing biofilms were defined as the lowest concentration of peptide needed to reduce metabolic activity, measured by an XTT assay, of the cells in biofilms to less than 10% compared to control untreated biofilms. We observed that the MICs of all β-peptides against biofilms were in the range of 128 to > 512 µg/mL and in many cases were much higher than corresponding planktonic MICs (Table 2, Table S4). Increasing the hydrophobicity of the β-peptide did not decrease the MIC against pre-formed *C. albicans* biofilms (Figure 5B). However, β-peptides **9**–**16** containing the N-terminal β^3^-hTyr residue had higher MICs compared to peptides **1**–**8**, which did not contain the β^3^-hTyr residue (Figure 5B). 

### 2.6. Hydrophobicity of β-Peptide Affects Prevention of C. albicans Biofilm Formation

While β-peptides exhibited low activities against existing biofilms, the ability to prevent biofilm formation would be of practical utility. To determine if our β-peptides could inhibit biofilm formation, we quantified the formation of *C. albicans* biofilms in the presence and absence of β-peptides **1**–**16**. 1000-Fold higher cell concentrations compared to planktonic susceptibility experiments were added to 2-fold dilutions of β-peptide and incubated at 37 °C to form biofilms for 48 h. XTT was used to measure the metabolic activities of the cells and determine the MIC of the peptide against biofilm formation, defined as the lowest concentration of the peptide that resulted in metabolic activity of 10% or less compared to untreated control cells (Figure S4). β-Peptides exhibited MICs in the range of 4–512 µg/mL (Table S4) and all β-peptides that were active against planktonic *C. albicans* were also effective in preventing biofilm formation to similar extents (Figure 5C and Table 2, Table S4). Additionally, we observed β-peptide hydrophobicity to be an important design parameter for preventing biofilm formation, with β-peptides having greater hydrophobicity being more effective in preventing *C. albicans* biofilm formation (Figure 5D). The correlation between hydrophobicity and anti-biofilm-forming activity occurred independent of other structural features of the β-peptides, such as the identity of the cationic residue (β^3^-hLys or β^3^-hArg) and the N-terminus (with or without β^3^-hTyr). 

### 2.7. β-Peptides Prevent C. glabrata, C. parapsilosis and C. tropicalis Biofilm Formation 

**Figure 6:**
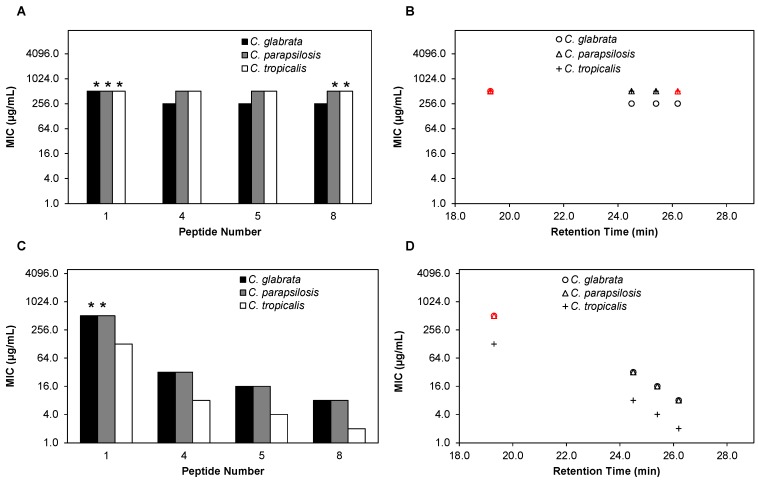
Antifungal activities of β-peptides against *C. glabrata, C. parapsilosis* and *C. tropicalis* biofilms. (**A**) Mature biofilms were formed by incubating *C. glabrata* (black bars)*, C. parapsilosis* (grey bars), and *C. tropicalis* (white bars) cells (10^6^ cells/mL) for 48 h. Planktonic cells were removed, biofilms were washed, and 2-fold dilutions of β-peptides were added and incubated for an additional 48 h. β-Peptide MICs were quantified using an XTT assay to compare the absorbance at 490 nm for β-peptide-treated samples and untreated samples. (**B**) Plot of biofilm MICs versus RP-HPLC retention times of β-peptides **1**, **4**, **5** and **8** against *C. glabrata* (circles)*, C. parapsilosis* (triangles), and *C. tropicalis* (plus signs). (**C**) MICs against *C. glabrata* (black bars)*, C. parapsilosis* (grey bars), and *C. tropicalis* (white bars) biofilm formation were determined by incubating cells (10^6^ cells/mL) with 2-fold dilutions of β-peptides for 48 h. Planktonic cells were removed, biofilms were washed, and β-peptide susceptibility was assessed using an XTT assay to compare the absorbance at 490 nm for β-peptide-treated samples and untreated samples. (**D**) Plot of MICs for inhibition of biofilm formation versus RP-HPLC retention times of the β-peptides **1**, **4**, **5** and **8** against *C. glabrata* (circles)*, C. parapsilosis* (triangles), and *C. tropicalis* (plus signs)*.* Experiments were performed on at least two independent days with three replicates each. Asterisks (*) and red symbols indicate that MIC was > 512 µg/mL, the highest concentration of peptide assayed.

*C. glabrata*, *C. parapsilosis*, and *C. tropicalis* are all species capable of forming biofilms to varying extents and biofilm formation is a virulence factor responsible for their pathogenicity [32,37,38]. We therefore also evaluated whether β-peptides were effective in disrupting or preventing biofilms of these species. We selected β-peptides **4**, **5** and **8** (that were active) and β-peptide **1** (that was inactive) against planktonic cells, and evaluated the MIC of these peptides against existing *C. glabrata*, *C. parapsilosis*, and *C. tropicalis* biofilms (Figure S5). As observed in *C. albicans* experiments, all β-peptides exhibited substantially greater MICs against pre-formed biofilms compared to planktonic cells (Table S5). Additionally, there was not a correlation between hydrophobicity of the β-peptide and MIC observed against biofilms (Figure 6A, B). However, we found that the MICs of β-peptide required in preventing biofilms were similar to the planktonic MICs for all β-peptides and species tested (Figure 6C, Figure S6 and Figure 2A). Further, the MICs of the β-peptides observed against all the *Candida* species were inversely correlated to the hydrophobicity of the β-peptide (Figure 6D). 

## 3. Discussion

For AMPs and their structural mimetics to realize their potentials as antimicrobial therapeutics, prevention of biofilm formation is an important criterion. 14-Helical β-peptides form well-characterized secondary structures and can be designed to possess desired properties by sequence-specific incorporation of β-amino acid residues [50,51,52]. Prior studies have demonstrated that globally-amphiphilic β-peptides can exhibit specific activity against *C. albicans* [30]. Here, we evaluated the antifungal activity of 14-helical β-peptides against multiple *C. albicans* clinical isolates, including a drug-resistant strain, and against other pathogenic *Candida spp*. We further evaluated the effect of β-peptides on biofilms of these species and identified a correlation between β-peptide hydrophobicity and antifungal activity against both planktonic cells and during prevention of biofilm formation. 

Planktonic susceptibility experiments against three *C. albicans* clinical isolates indicated that β-peptides are effective against multiple strains and that hydrophobicity correlates with β-peptide activity against *C. albicans*. It is particularly noteworthy that even though the K1 strain is fluconazole-resistant, it is still susceptible to the action of β-peptides, indicating that the membrane-disruption mechanism of action of these β-peptides is effective even against *C. albicans* strains that acquire resistance to traditional antifungal drugs. Although the mechanism of action of many AMPs in general, and β-peptides in particular, is thought to involve cell membrane disruption [30,48,49], there is limited understanding regarding the role that the cell wall plays. The cell wall has been suggested to be necessary for the activity of AMPs in certain cases and plays an important role in the capture and anchoring of peptides to the cell [49,53,54]. In other instances, the cell wall has been shown to decrease the activity of AMPs by sequestering the peptide and acting as a barrier to the translocation of peptide into the cell [49,55]. We observed that the planktonic MICs of both active and inactive β-peptides did not change upon removing the cell wall from *C. albicans* yeast indicating that the cell wall does not affect the antifungal action of β-peptides. 

The hydrophobicity of both antimicrobial α-peptides and 14-helical β-peptides has been shown to be important for antifungal activity against planktonic *C. albicans* cells, but studies indicate that AMPs that are active against planktonic cells need not necessarily be active against biofilm cells (and *vice versa*) [31,40,41,56]. We used a simplified *in vitro* biofilm model to study the effects of β-peptide in preventing the formation of and inhibiting existing biofilms of various *Candida spp.* Although *in vitro* biofilms develop and exist in a different context from those that occur *in vivo*, where shear force and continuous flux of material play important roles in biofilm development, *in vitro* static biofilms provide a good starting point to understand the effects of β-peptide on biofilms. Our results indicate that the antifungal MICs of 14-helical β-peptides against planktonic *C. albicans* (and in preventing biofilm formation) depend on the hydrophobicity of the β-peptide: more hydrophobic peptides exhibit greater antifungal activities, with lower MICs. However, the MICs of the β-peptides against existing biofilms were higher than those against planktonic cells or for inhibiting biofilm formation, and were also independent of β-peptide hydrophobicity. Mature *C. albicans* biofilms consist of communities of both yeast and hyphal cells encased in a three-dimensional extracellular matrix (ECM), whereas during the process of biofilm formation cells are mainly in the yeast form with little or no ECM [38,57]. We hypothesize that the higher MICs observed against biofilms could potentially be due to multiple reasons including (i) the increase in concentration of the cells in the biofilm [57], leading to peptide potentially being sequestered onto the cell membrane, thus depleting free peptide remaining in solution (however, it is noteworthy that all β-peptides have nearly the same MICs against planktonic cells and during inhibition of biofilm formation, even though the cell concentration was 1000-fold higher in the latter experiment), (ii) differences in the phenotype and morphology of the cells present in the mature biofilm compared to planktonic cells [18] and their differential susceptibility to β-peptides, and (iii) biofilm ECM trapping the β-peptide and making them unavailable in sufficient amounts to lyse *C. albicans* cells. Studies have demonstrated that components of the ECM, including glycans, are capable of sequestering traditional antifungal drugs and are responsible for increased MICs [58]. Nevertheless, from a translational standpoint it is important to note that β-peptides remain active against biofilm formation and therefore have potential for prophylaxis and early-stage infection prevention. 

Other *Candida* species, especially *C. glabrata*, *C. parapsilosis*, and *C. tropicalis*, have increasingly been observed as infectious agents in hospital settings and therefore there is a growing need to develop drugs or test existing drugs to combat them [33]. Some of the drugs used against *C. albicans*, including amphotericin B and fluconazole, are also used to treat *C. glabrata*, *C. parapsilosis*, and *C. tropicalis* infections [37]. However, few AMPs have been tested against these *Candida* species [37,45,46,47]. We found that 14-helical β-peptides are also active against *C. glabrata*, *C. parapsilosis*, and *C. tropicalis* species to varying extents against planktonic cells and in preventing biofilm formation. Additionally, we observed that even at sub-MIC concentrations, the fungi were affected by the peptide and we observed stunted pseudohyphae and more rounded yeast, especially in *C. tropicalis*, similar to what has been previously reported for *C. albicans* [59] (Figure S3). Among planktonic cells, we observed that *C. tropicalis* was the most susceptible to β-peptides while *C. glabrata* was the least susceptible. *C. glabrata* is known to exhibit higher MICs against traditional antifungal drugs and against cationic antifungal peptides [34,60]. Here, differences in membrane composition may potentially explain the variation in susceptibility of each species to the β-peptides investigated here [61]. Despite *C. glabrata* being the most resistant to the planktonic antifungal activity of β-peptides, we observed that *C. glabrata* biofilms were more susceptible to β-peptides compared to *C. parapsilosis* and *C. tropicalis* biofilms. This is possibly due to the fact that *C. glabrata* form less robust biofilms in comparison to the other two *Candida* species [32]. The direct relationship between the hydrophobicity of β-peptide and its antifungal activity observed against planktonic *C. albicans* was also observed against planktonic *C. glabrata*, *C. parapsilosis*, and *C. tropicalis*. 

## 4. Experimental Section 

### 4.1. Materials

Fmoc-β-amino acids, including Fmoc-l-β-homoalanine, Fmoc-l-β-homovaline, Fmoc-*O*-tert-butyl-l-β-homotyrosine, *N*β-Fmoc-Nω-Boc-l-β-homolysine, and Fmoc-*N*ω-(2,2,5,7,8-pentamethyl-chromane-6-sulfonyl)-l-β-homoarginine were purchased from Chem-Impex International, Inc. (Wood Dale, IL, USA) TentaGel S RAM Fmoc, HBTU (*O*-(benzotriaole-1-yl)-*N,N,N',N'-*tetramethyluronium hexafluorophosphate), and HOBt^.^H_2_O (*N*-hydroxybenzotrizole monohydrate) were purchased from Advanced ChemTech. (Louisville, KY, USA) (*S*)-3-Aminopentanoic acid was purchased from Sigma-Aldrich (St Louis, MO, USA) for synthesis of Fmoc-(*S*)-3-aminopentanoic acid (Fmoc-β^3^-Et-OH). RPMI 1640 powder (with l-glutamine and phenol red, without HEPES and sodium bicarbonate) and 2,3-bis-(2-methoxy-4-nitro-5-sulfophenyl)-2*H*-tetrazolium-5-carboxanilide (XTT) were purchased from Invitrogen (Grand Island, NY, USA). 3-(*N*-Morpholino) propanesulfonic acid (MOPS) and phosphate-buffered saline (PBS) liquid concentrate (10X) were purchased from Fisher Scientific (Pittsburgh, PA, USA). Zymolase was purchased from Zymo Research (Irvine, CA, USA). Menadione and melittin were purchased from Sigma-Aldrich.

### 4.2. β-Peptide Synthesis

β-peptides were synthesized using TentaGel (20–40 µmol) microwave-assisted solid phase peptide synthesis procedures similar to those reported previously [62]. Briefly, the solution of Fmoc-β-amino acid, coupling reagent (HBTU, HOBt), and base (DIPEA) in DMF were mixed before coupling. Microwave (CEM Discover) irradiation methods were used for coupling of Fmoc-β-amino acid (600 W maximum power, 70 °C, ramp 2 min, hold 12 min) and deprotection of Fmoc (600 W maximum power, 80 °C, ramp 2 min, hold 6 min). After each coupling and deprotection step, the resin was thoroughly washed with DMF and CH_2_Cl_2_, and then the peptide was cleaved from the resin by TFA containing H_2_O (2.5%) and triisopropylsilane (2.5%) for 1 to 2 h. The crude product was purified by preparative RP-HPLC with a gradient of 25%–73% CH_3_CN in water containing 0.1% TFA.

### 4.3. Characterization of β-Peptide Hydrophobicity 

Hydrophobicity of 14-helical β-peptides was characterized by measuring retention times with an analytical RP-HPLC using a C18 column (Waters, X-bridge, column dimensions 4.6 mm × 250 mm, Milford, MA, USA). The β-peptide solutions (0.5 to 1 mg/mL, 50 µL) were injected into the HPLC at a flow rate of 1 mL/minute. Retention time was characterized in triplicate with a gradient of 20%–80% CH_3_CN in water containing 0.1% TFA over 5–35 min.

### 4.4. Yeast Strain and Culture Conditions

*C. albicans* strains SC5314 and ATCC 90028 were purchased from ATCC. *C. albicans* K1, *C. glabrata* 5376, *C. parapsilosis* 5986 and *C. tropicalis* 98-234 are clinical isolates from invasive candidiasis [63] and were generously donated by the Andes group (University of Wisconsin-Madison). K1 is fluconazole resistant [42,43]. All strains were stored as a 50% glycerol stock at −80 °C and grown in a liquid YPD media. For planktonic susceptibility testing, cells were grown on agar plates at 30 °C for 24 h and 2–3 colonies were used for the cell inoculum. For spheroplast formation and biofilm susceptibility testing assays, cells were grown overnight in YPD at 30 °C. RPMI (with l-glutamine and phenol red buffered with 3-(*N*-morpholino) propanesulfonic acid and adjusted to pH 7.0) was the media used for all susceptibility tests. XTT solution for assessing metabolic activity was prepared at 0.5 g/L in PBS with 3 μM menadione in acetone and pH adjusted to 7.4. 

### 4.5. Planktonic Antifungal Susceptibility Testing

The antifungal activities of the β-peptides against *C. albicans, C. glabrata, C. parapsilosis, C. tropicalis* cells were assayed in accordance to the guidelines for planktonic susceptibility testing provided by the Clinical and Laboratory Standards Institute, but with a modification to include a quantitative XTT cell metabolic activity end-point [30]. 100 µL of two-fold serially diluted concentrations of β-peptides in RPMI were mixed with 100 μL of a *C. albicans* strain SC5314, ATCC 90028, K1, *C. glabrata* 5376, *C. parpsilosis* 5986, *C. tropicalis* 98-234 cell suspensions adjusted to 1× 10^3^ –5 × 10^3^ cells/mL and the plates were incubated at 35 °C for 48 h. Cell only controls consisting of wells without β-peptide and media controls consisting of wells without both β-peptide and cells were included in the 96-well plate, for every β-peptide screened. After 48 h, the MICs were recorded visually as the first well that had growth and by using an XTT metabolic assay. 

### 4.6. Fluorescence Imaging of Planktonic C. glabrata, C. parapsilosis and C. tropicalis 

To image the interaction of peptide **4_FL_** with *C. glabrata*, *C. parapsilosis* and *C. tropicalis* cells, a cell suspension of 1 × 10^5^ cells/mL was prepared in RPMI 1640. Dilutions of peptide **4_FL_**_,_ a positive kill control with methanol, and a β-peptide-free control were prepared, and 50 µL of each solution was added to an equal volume of cell suspension. Cells were incubated with the solutions for 3.5 h at 37 °C, PI was added at a final concentration of 1 µg/mL and plates were incubated at 37 °C for an additional 45 min. Cells were imaged using a fluorescence microscope with a red filter for PI and a blue filter for peptide **4_FL_**.

### 4.7. C. albicans Spheroplast Formation and Characterization

*C. albicans* cells from overnight cultures (1 mL) in YDP were pelleted and resuspended in 1 mL of spheroplasting buffer (0.1 M sodium phosphate, 1.2 M sorbitol, 0.05 M EDTA adjusted to pH 8.0) and 3.5 µL of β-mercaptoethanol and incubated for 15 min at room temperature. The solution was pelleted and resuspended in 300 µL of spheroplasting buffer and 4.5 µL of zymolase and incubated at 37 °C for 2 h, after which the supernatant was removed and sphroplasts were resuspended in spheroplasting buffer. Spheroplasts were characterized visually by microscopy. Additionally, increasing concentrations of SDS were added to yeast and spheroplast cells and the cells were plated on YPD agar plates to assess cell viability after growth overnight at 37 °C. Spheroplasts were used immediately for planktonic susceptibility experiments. 

### 4.8. Antifungal Biofilm Susceptibility Testing 

The antifungal activities of the β-peptides against *Candida* biofilms were assayed by a method similar to a previously reported protocol [30]. Briefly, overnight cultures of *C. albicans* SC5314, *C. glabrata*, *C.* *parapsilosis*, and *C. tropicalis* cells were washed with PBS and re-suspended in RPMI. 100 μL of cell suspension adjusted to of 10^6^ cells/mL with RPMI 1640 was added to wells of 96-well plates and were statically incubated at 37 °C for 48 h to allow biofilm formation. After 48 h of biofilm formation, biofilms were washed twice with PBS to remove non-adherent cells and 100 μL of two-fold serial dilutions of β-peptides in RPMI were added to the biofilms and plates were incubated at 37 °C for an additional 48 h. Biofilms were washed with PBS and XTT was used to quantify metabolic activity of biofilms. 

### 4.9. Biofilm Formation in the Presence of β-Peptides 

*C. albicans*, *C. glabrata*, *C. tropicalis*, and *C. parapsilosis* biofilm formation in the presence of β-peptide was evaluated using a procedure used previously [30]. Briefly, 100 μL of overnight cultures of *C. albicans* SC5314, *C. glabrata*, *C. parapsilosis*, and *C. tropicalis* adjusted to 2 × 10^6^ cells/mL was added to 100 μL of two-fold serially diluted β-peptide in a 96-well plates. Peptide-free controls and cell and peptide-free controls were included for each peptide assayed and the plates were incubated at 37 °C for 48 h. After 48 h, biofilms were washed with PBS to remove non-adherent cells and the metabolic activity of biofilms grown in the presence of β-peptide was determined using XTT.

### 4.10. Quantification of Cell Metabolic Activity Using an XTT Assay 

For evaluation of metabolic activities of planktonic cells and biofilms in susceptibility testing in 96-well plates, 100 μL of XTT solution was added to every well and plates were incubated at 37 °C in the dark for 1.5 h for *C. albicans* and 2.5 h for *C. glabrata*, *C. parapsilosis* and *C. tropicalis*. At the end of the incubation, 75 μL of supernatant from wells were transferred to a new plate and absorbance at 490 nm was measured. The percent metabolic activity was calculated as:

$$Metabolic\;Activity\;\left(\%\right)=\;\frac{\left(A_{490}-\;A_{490}^{background}\right)}{A_{490}^{cell\;control}-\;A_{490}^{background}}\;\times 100$$

where $A_{490}$, $A_{490}^{cell\;control}$, and $A_{490}^{background}$ are the average absorbance values of wells containing a specific concentration of β-peptide, cell control wells without β-peptide, and media only wells, respectively. Experiments were performed in triplicate and repeated on at least two different days. Metabolic activity was plotted as a function of β-peptide concentration and the lowest assayed concentration of β-peptide that resulted in less than a 10% average metabolic activity of planktonic cells was taken as the planktonic minimum inhibitory concentration (MIC) of that β-peptide. Similarly, MICs against existing biofilms and for inhibition of biofilm formation represent the lowest concentrations of β-peptide that resulted in less than 10% average metabolic activity of biofilm cells, determined by an XTT assay, normalized to metabolic activity from corresponding untreated control biofilms cultured in a similar manner.

## 5. Conclusions

We have demonstrated that 14-helical β-peptides are active against multiple clinical isolates of *C. albicans*, including a drug-resistant strain, and other opportunistic *Candida spp.* such as *C. glabrata*, *C. parapsilosis*, and *C. tropicalis*. The antifungal activities of β-peptides directly correlated to the hydrophobicities of the peptides for all strains and species. Additionally, we demonstrated that while the activities of these β-peptides against existing *Candida* biofilms were low, they prevented *Candida spp.* from forming biofilms. The hydrophobicity of the β-peptides is an important design criterion in biofilm prevention, with more hydrophobic peptides resulting in greater inhibition of biofilm formation. The results of this study demonstrate that globally-amphiphilic hydrophobic β-peptides can prevent multiple *Candida* species from forming biofilms, indicating the therapeutic potential of this class of compounds in preventing fungal infections. 

## Supplementary Materials

Supplementary materials can be accessed at: http://www.mdpi.com/1424-8247/8/3/483/s1.
