# Supplementary material for: Antifungal Activity of 14-Helical β-Peptides against Planktonic Cells and Biofilms of Candida Species
Source: Pharmaceuticals (Basel). 2015 Aug 13;8(3):483–503. doi: 10.3390/ph8030483 (PMC4588179; doi:10.3390/ph8030483)
Supplement: Supplementary file 1 [file pharmaceuticals-08-00483-s001.pdf]

Supplementary

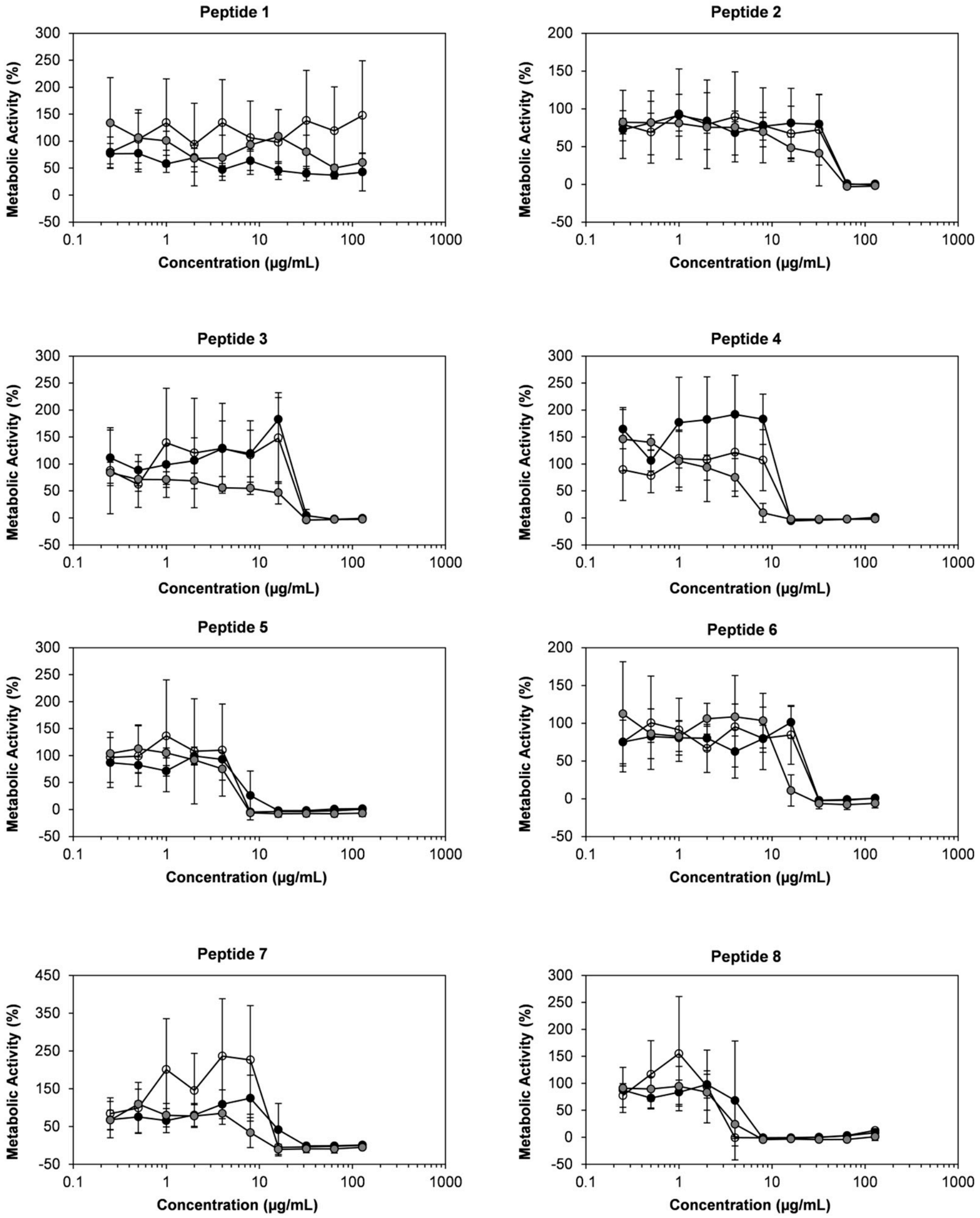

Figure S1. Plots of concentration-dependent planktonic growth inhibition of different strains of *C. albicans* by β-peptides **1**-**8**. *C. albicans* ATCC 90028 (black circles), SC5314 (grey circles), and K1 (white circles) cells (10^3 cells/mL) were incubated with β-peptides for 48 hours and β-peptide susceptibility was assessed using an XTT assay to compare the absorbance at 490 nm for β-peptide-treated samples and untreated samples. Y-axes represent metabolic activity normalized to the untreated control. Data points are the average of two independent experiments of three replicates each and error bars denote standard deviation.

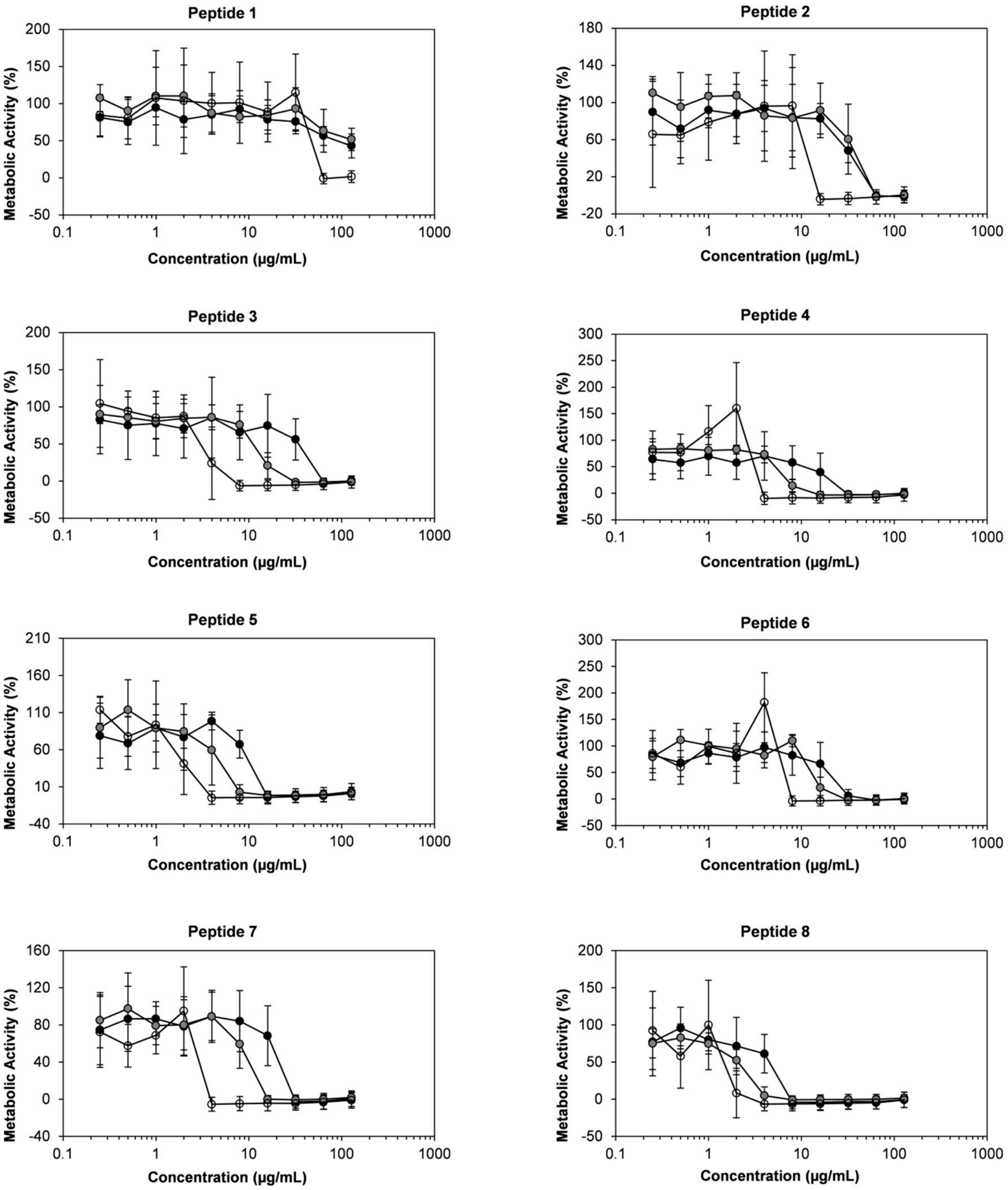

Figure S2. Plots of concentration-dependent planktonic growth inhibition of different pathogenic *Candida* species by β-peptides **1**-**8**. *C. glabrata* (black circles), *C. parapsilosis* (grey circles), and *C. tropicalis* (white circles) cells (10^3 cells/mL) were incubated with β-peptides for 48 hours and β-peptide susceptibility was assessed using an XTT assay to compare the absorbance at 490 nm for β-peptide-treated samples and untreated samples. Y-axes represent metabolic activity normalized to the untreated control. Data points are the average of two independent experiments of three replicates each and error bars denote standard deviation.

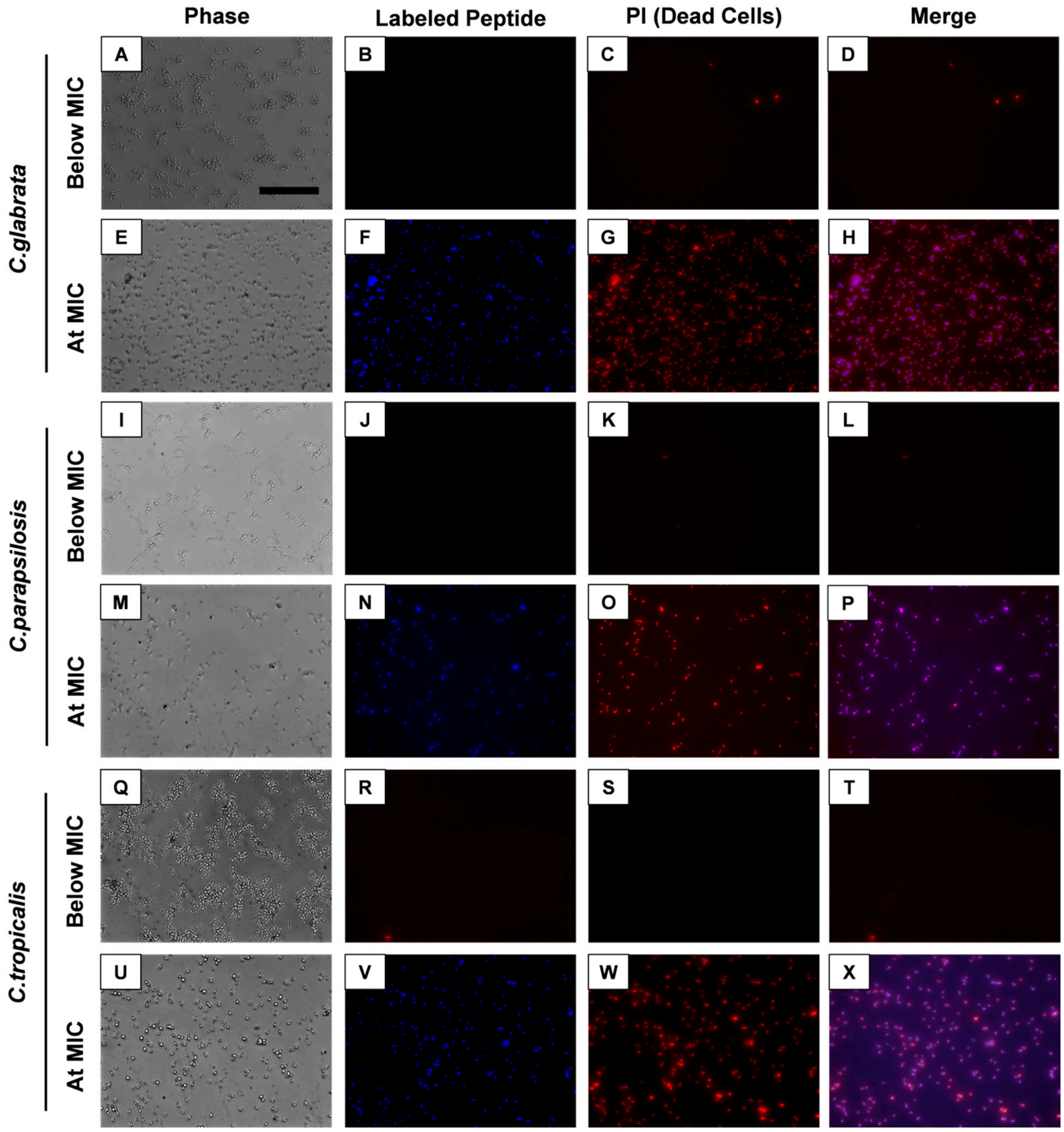

Figure S3. Phase contrast and fluorescence micrographs of *C. glabrata* (A-H), *C. parapsilosis* (I-P), and *C. tropicalis* (Q-X) treated with $\mathbf{4}_{\mathrm{FL}}$.Cells (10^5 cells/mL) were treated with the $\mathbf{4}_{\mathrm{FL}}$(blue) at a concentration 4-fold below the MIC (A-D, I-L, Q-T) and at MIC (E-H, M-P, U-X) for 3.5 h. Cells were stained with PI to identify dead cells (red). Scale bar = 100 μm.

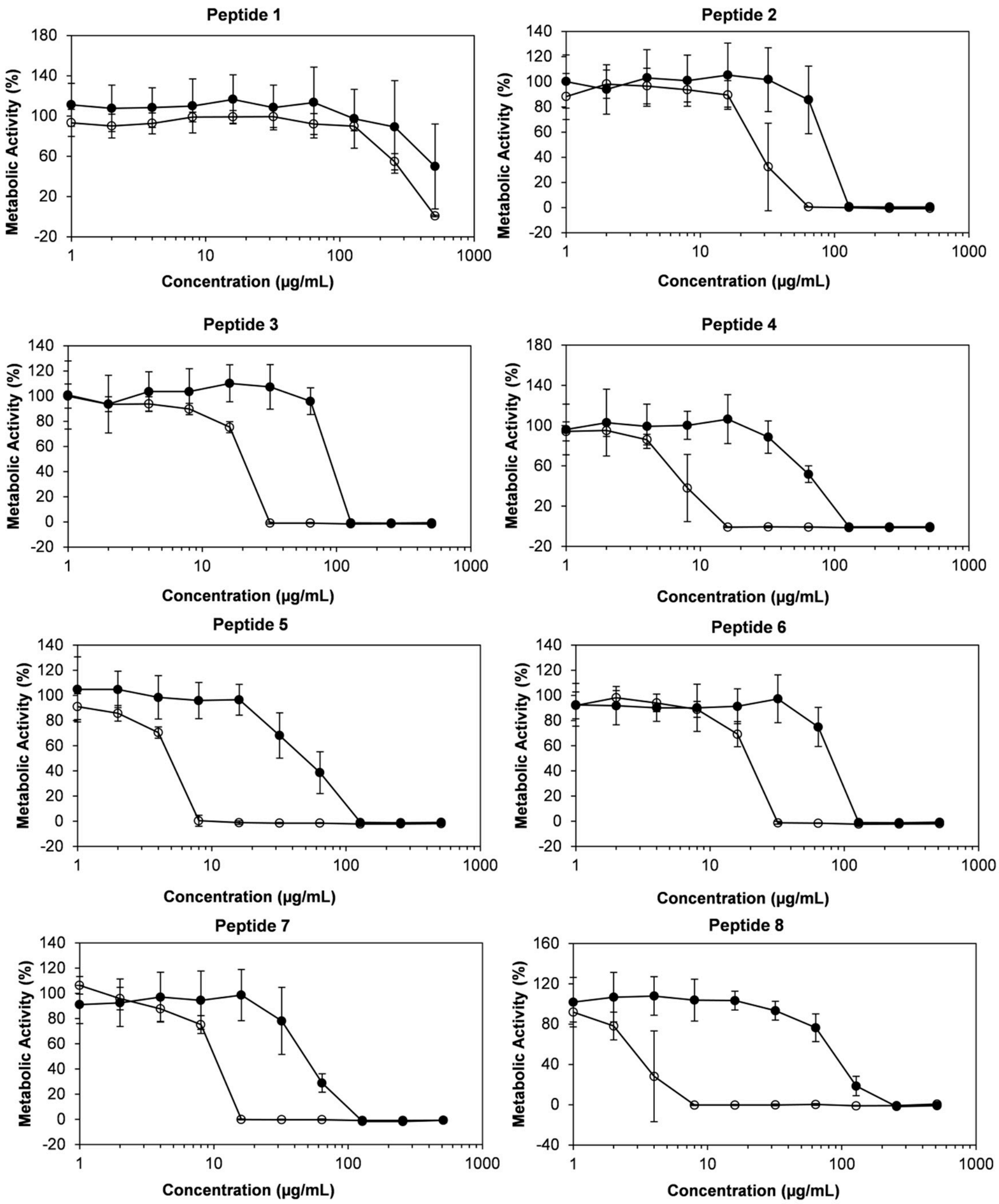

Figure S4. *Cont.*

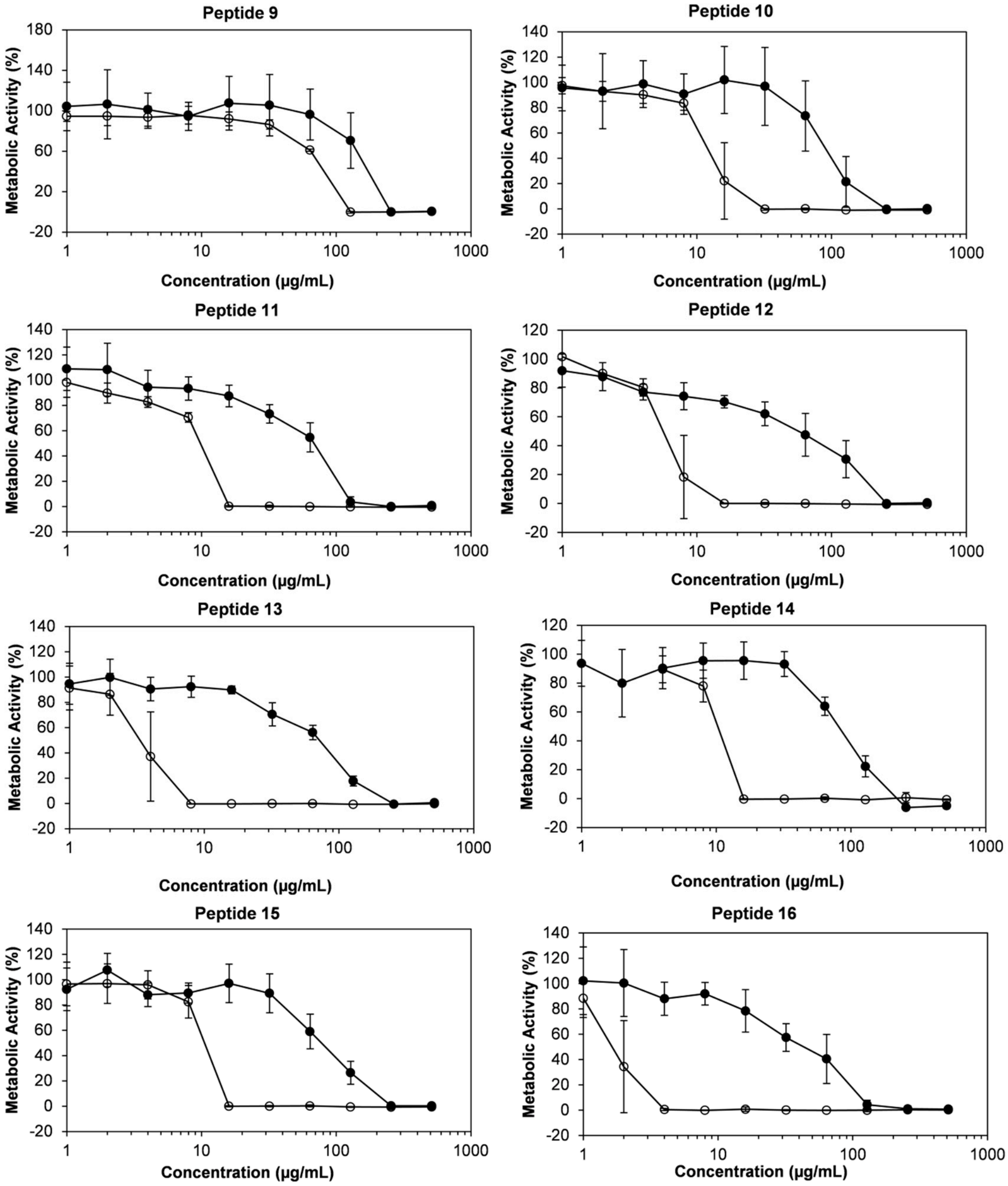

Figure S4. Plots of concentration-dependent biofilm inhibition of *C. albicans* by β-peptides **1-16**. β-peptides were added to freshly prepared *C. albicans* cells (10^6 cells/mL) or 48 hours mature *C. albicans* biofilms and grown for 48 hours at 37 °C to evaluate extent of biofilm formation prevention (white circles) and pre-formed biofilm inhibition (black circles) respectively. β-Peptide susceptibility was assessed using an XTT assay to compare the absorbance at 490 nm for β-peptide-treated samples and untreated samples. Y-axes represent metabolic activity normalized to the untreated control. Data points are the average of two independent experiments of three replicates each and error bars denote standard deviation.

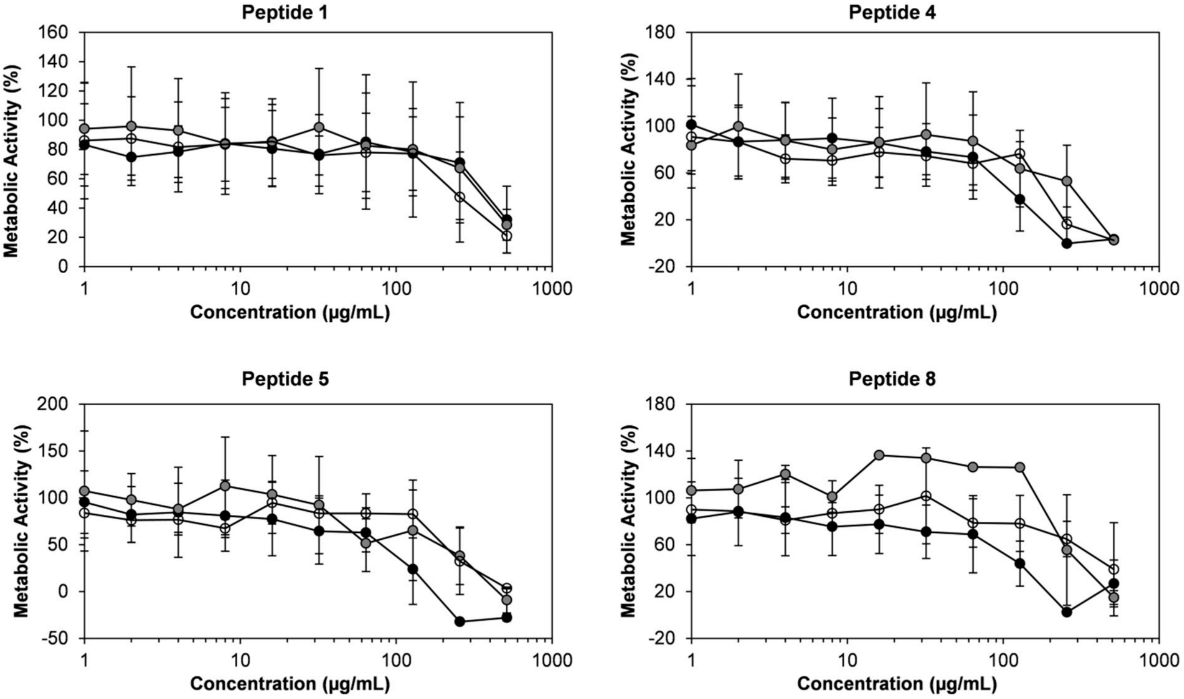

Figure S5. Plots of concentration-dependent pre-formed biofilm inhibition of other pathogenic *Candida* species by β-peptides **1**, **4**, **5**, and **8**. *C. glabrata* (black circles), *C. parapsilosis* (grey circles), and *C. tropicalis* (white circles) cells (10^6 cells/mL) were grown for 48 h and then incubated with β-peptides for a further 48 hours and β-peptide susceptibility was assessed using an XTT assay to compare the absorbance at 490 nm for β-peptide-treated samples and untreated samples. Y-axes represent metabolic activity normalized to the untreated control. Data points are the average of two independent experiments of two replicates each and error bars denote standard deviation.

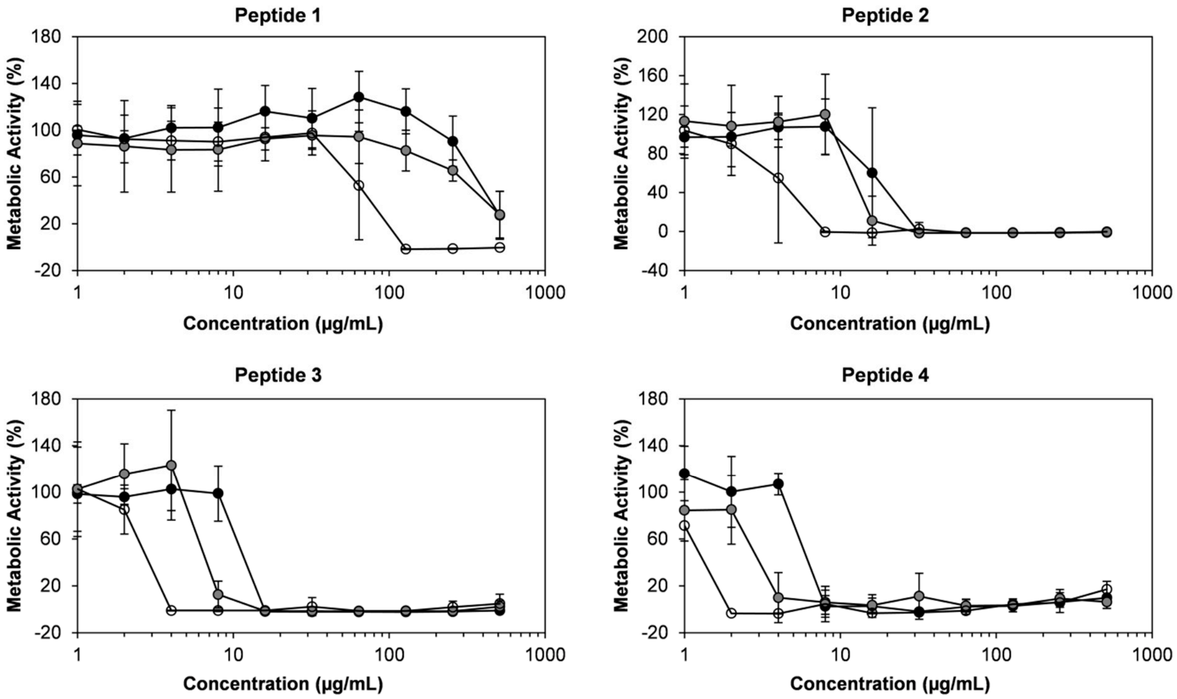

Figure S6. Plots of concentration-dependent biofilm formation inhibition of other pathogenic *Candida* species by β-peptides **1**, **4**, **5**, and **8**. *C. glabrata* (black circles), *C. parapsilosis* (grey circles), and *C. tropicalis* (white circles) cells (10^6 cells/mL) were incubated in the presence of β-peptides for 48 hours and β-peptide susceptibility was assessed using an XTT assay to compare the absorbance at 490 nm for β-peptide-treated samples and untreated samples. Y-axes represent metabolic activity normalized to the untreated control. Data points are the average of two independent experiments of two replicates each and error bars denote standard deviation.

Table S1. Hemolysis of β-peptide **1-16**, concentration of β-peptide needed for 50% lysis of human red blood cells (hRBCs)

Peptide #	Peptide Concentration at 50% Hemolysis a (HC50, μg/mL)
1	>400
2	>400
3	>400
4	161 ± 40
5	77 ± 16
6	>400
7	341 ± 34
8	15 ± 7
9	>400
10	149 ± 20
11	79 ± 8
12	12 ± 6
13	11 ± 2
14	106 ± 37
15	73 ± 40
16	3 ± 1

[a] Hemolysis assays were performed as previously indicated in Lee, M.R. et al., *ACS Chem. Biol.* (2014). Briefly, equal volumes of 2-fold dilutions of β-peptides in tris-buffered saline (TBS) were incubated with hRBCs for an hour at 37 °C. Supernatants were transferred to a new plate and absorbance was measured at 405 nm. % hemolysis was calculated relative to positive lysis control and the concentration of peptide needed for 50% hemolysis was recorded as HC_{50} for that peptide. Values reported are average measurements from three independent experiments of duplicates each and the error denoted is the standard deviation. Concentration dependent hemolysis graphs can be found in Lee, M.R. *et al.*, *ACS Chem. Biol.* (2014).

Table S2. Minimum inhibitory concentrations (MICs) of peptide **1-8** against *C. glabrata*, *C. parapsilosis*, and *C. tropicalis.*

**Peptide #**	**MIC [a] (μg/mL)**		
	C. glabrata	***C. parapsilosis***	***C. tropicalis***
**1**	> 128	> 128	64
**2**	64	64	16
**3**	64	32	8
**4**	32	16	4
**5**	16	8	4
**6**	32	32	8
**7**	32	16	4
**8**	8	4	2

[a] MICs were determined by incubating *C. glabrata*, *C. parapsilosis*, and *C. tropicalis* cells (10^3 cells/mL) with β-peptides for 48 hours and β-peptide susceptibility was assessed using an XTT assay to compare the absorbance at 490 nm for β-peptide-treated samples and untreated samples.

Table S3. Minimum inhibitory concentrations (MICs) of peptide **4** and **4$_{FL}$** against *C. glabrata*, *C. parapsilosis*, and *C. tropicalis*.

***Candida* Species**	**MIC of Peptide 4 [a] (μg/mL)**	**MIC of Labeled Peptide 4$_{FL}$ [a] (μg/mL)**	***Candida* Species**
C. glabrata	32	32	*C. glabrata*
C. parapsilosis	16	16	*C. parapsilosis*
C. tropicalis	4	8	*C. tropicalis*

[a] MICs were determined by incubating *C. glabrata*, *C. parapsilosis*, and *C. tropicalis* cells (10^3 cells/mL) with β-peptides for 48 hours and β-peptide susceptibility was assessed using an XTT assay to compare the absorbance at 490 nm for β-peptide-treated samples and untreated samples.

Table S4. Minimum inhibitory concentrations (MICs) of peptide **1-16** against *C. albicans* biofilms.

**Peptide #**	**MIC [a] (μg/mL)**	
	**Pre-Formed Biofilms**	**Biofilm Formation**
1	>512	512
2	128	64
3	128	32
4	128	16
5	128	8
6	128	32
7	128	16
8	256	8
9	256	128
10	256	32
11	128	16
12	256	16
13	256	8
14	256	16
15	256	16
16	128	4

Table S5. Minimum inhibitory concentrations (MICs) of peptide **1**, **4**, **5**, and **8** against *C. glabrata, C. parapsilosis*, and *C. tropicalis* biofilms.

Peptide #	Pre-Formed Biofilm MIC [a] (μg/mL)			Biofilm Formation MIC [a] (μg/mL)		
	C. glabrata	***C. parapsilosis***	***C.tropicalis***	***C. glabrata***	***C. parapsilosis***	***C.tropicalis***
1	>512	>512	>512	>512	>512	128
4	256	512	512	32	32	8
5	256	512	512	16	16	4
8	256	>512	>512	8	8	2

[a] For determining MICs against pre-formed biofilms, β-peptides were added to 48 hours mature *C. glabrata*, *C. parapsilosis*, and *C. tropicalis* biofilms and grown for 48 hours at 37 °C. For biofilm formation MICs, β-peptides were added to freshly prepared *C. glabrata*, *C. parapsilosis*, and *C. tropicalis* cells (10^6 cells/mL) and grown for 48 hours at 37 °C. β-Peptide susceptibility was assessed using an XTT assay to compare the absorbance at 490 nm for β-peptide-treated samples and untreated samples.
